# Effects of depot-medroxyprogesterone acetate on the immune microenvironment of the human cervix and endometrium: implications for HIV susceptibility

**DOI:** 10.1038/mi.2016.121

**Published:** 2017-01-04

**Authors:** K K Smith-McCune, J F Hilton, U Shanmugasundaram, J W Critchfield, R M Greenblatt, D Seidman, S Averbach, L C Giudice, B L Shacklett

**Affiliations:** 1 Department of Obstetrics, Gynecology and Reproductive Sciences, University of California, San Francisco, California, USA; 2 Department of Epidemiology and Biostatistics, University of California, San Francisco, California, USA; 3 Department of Medical Microbiology and Immunology, School of Medicine, University of California, Davis, California, USA; 4 Departments of Clinical Pharmacy and Medicine, University of California, San Francisco, California, USA

## Abstract

Depot-medroxyprogesterone acetate is a commonly used injectable contraceptive that has been associated with an increased risk of HIV acquisition. This study compares effects of depot-medroxyprogesterone acetate on immune parameters from several upper reproductive tract compartments relevant to HIV-1 susceptibility in repetitive samples from 15 depot-medroxyprogesterone acetate users and 27 women not on hormonal contraceptives. Compared with samples from unexposed women in the mid-luteal phase, depot-medroxyprogesterone acetate use was associated with: increased endocervical concentrations of MCP1 and IFNalpha2; decreased endocervical concentrations of IL1beta and IL6; increased proportions of endometrial CD4+ and CD8+ cells expressing the activation marker HLADR; increased density of endometrial macrophages; and decreased density of endometrial regulatory T cells. Unlike previous reports with samples from the vagina, we did not observe increased expression of the HIV co-receptor CCR5 on CD4+ T cells in the endocervix or endometrium. Our results indicate important differences in anatomic compartments regarding mechanisms by which depot-medroxyprogesterone acetate could be associated with increased risk of HIV acquisition, including increased recruitment of macrophages to the endometrium, decreased levels of pro-inflammatory cytokines in the endocervix possibly leading to enhanced susceptibility to viral infection, and activation of endometrial T cells.

## Introduction

Forty-one million women worldwide use injectable hormonal contraception, over a quarter of whom live in regions of Africa where the overlap of injectable method use and HIV prevalence is striking.^1, 2^ With limited access to alternate contraceptive methods and antiretroviral therapy, many of these areas have high maternal and HIV-related mortality.^2^ One commonly used injectable contraceptive is depot-medroxyprogesterone acetate (DMPA). Two recent meta-analyses report that women using DMPA face a 40–50% greater risk of acquiring HIV.^1, 3^ The World Health Organization’s medical eligibility criteria for DMPA remains unchanged since 2012,^4^ although recent publications question these recommendations.^5^

DMPA may increase HIV susceptibility via a variety of mechanisms. Elevated progestin levels have been associated with: increased secretion of inflammatory mediators that recruit or activate immune target cells and/or facilitate HIV replication;^6^ decreased secretion of antimicrobial peptides that contribute to host defense;^7, 8^ increased co-receptor expression rendering cells more susceptible to HIV;^6, 9^ thinning of the epithelial barrier;^10^ changes in the vaginal microbiome;^10, 11^ increased genital herpes shedding;^12, 13^ increased risk of herpes acquisition;^14, 15^ and increased frequency of cervical HIV target cells (CCR5+ CD4 T cells).^16^ *In vitro* studies performed with activated peripheral blood mononuclear cell cultures showed that incubation with medroxyprogesterone acetate (MPA) increases HIV-1 replication and prevents downregulation of HIV co-receptors on CD4+ T cells.^9^ Immune cells from vaginal biopsies of women using DMPA also express high levels of CCR5.^6^ Finally, MPA treatment of immortalized endocervical cells results in reduced production of RANTES, a CCR5 ligand that can compete with HIV for CCR5 co-receptors and inhibit entry of the virus into the cell.^7^ To date, the majority of DMPA studies have been done with peripheral blood or vaginal specimens rather than with samples from the upper female reproductive tract (FRT), specifically the endocervix and uterus. These sites are relevant due to recent attention on the cervix and endometrium as potential portals of HIV entry.^17, 18, 19, 20, 21^

The purpose of this study was to examine the effects of DMPA on the immune microenvironment of the upper FRT, comparing biological samples from women using DMPA to those from control women not using hormonal or intrauterine contraception collected at the time of peak progesterone levels (6–10 days after ovulation). We compared immune parameters relevant to HIV-1 susceptibility in these samples, including endocervical chemokine/cytokine levels, T-cell phenotypes, and immune cell infiltrates.

## Results

### Demographics and DMPA exposure

Fifteen DMPA users and 27 Controls were included in the analysis (Table 1). DMPA users were younger than Controls (28 vs. 35 years) and more DMPA users identified as black (60 vs. 33%). At the time of sample collection, women had used DMPA for a median of 15 months (8–29 months, *n*=10; 30–108 months, *n*=4) and their median time since last DMPA injection was 29 days (30–102 days, *n*=6; 1–29 days, *n*=8). For Controls the median menstrual cycle day was 23, reflecting mid-luteal phase.

### Effects of DMPA on chemokines, cytokine and innate immune factors in the endocervical canal

**Figure 1:**
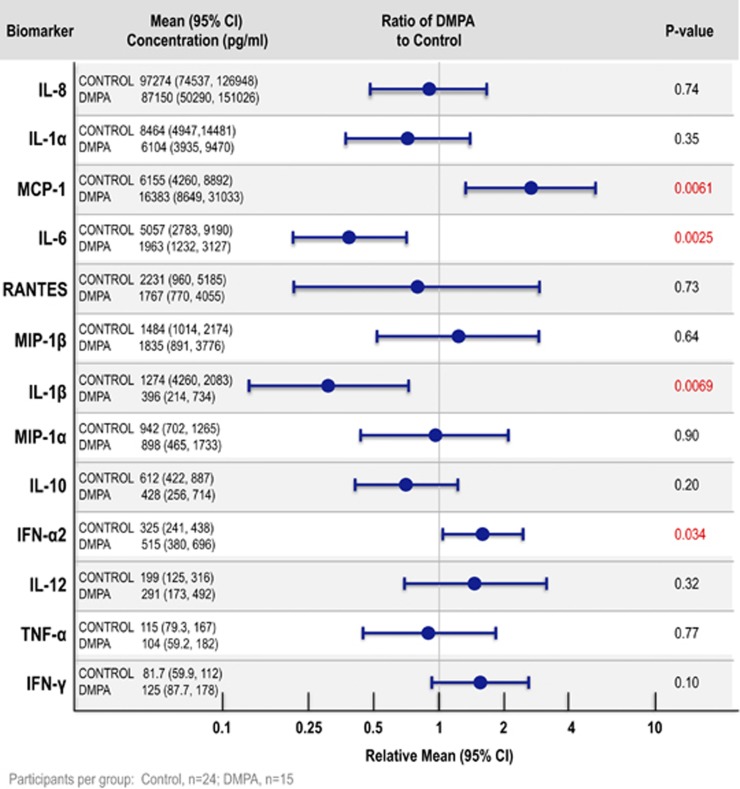
Comparisons of concentrations of 13 cytokines, chemokines and innate immune factors in endocervical fluids from Controls (*n*=24) and DMPA users (*n*=15). Fluids were collected by insertion of an ophthalmic sponge (Merocel) into the endocervical canal for 90 s; fluids were extracted and analyzed on a Milliplex platform as described in Methods section. The specific biomarker is indicated in the left column. The next column shows age-adjusted mean concentration (pg ml^−1^) and 95% confidence intervals of the corresponding biomarker from Controls and DMPA users, ordered by biomarker concentration in Controls from highest (top) to lowest (bottom). The middle column shows the ratio (circle) and 95% confidence intervals (bars) of concentrations from DMPA users compared with Control for each biomarker. The column in the far right gives specific *P*-values for the ratios with significant values (*P*-values <0.05) highlighted in red.

To determine whether DMPA altered the immune milieu of the endocervix, we studied age-adjusted concentrations of 13 proteins in endocervical fluid extracted onto absorbant wicks, contributed by 15 DMPA users and 24 Controls (Figure 1). The chemokine MCP1 was highly expressed in Controls, and was up-regulated in DMPA users (mean ratio, 2·66; 95% confidence interval (CI), 1·32–5·36). Levels of other chemokines (interleukin (IL)-8, MIP1alpha, MIP1beta, and RANTES) were not significantly different between DMPA users and Controls; however, higher age was statistically significantly (*P*<0.05) associated with lower expression of RANTES.

The age-adjusted expression level of IFNalpha2, an important component of the antiviral innate immune response at epithelial surfaces, was significantly up-regulated (mean ratio, 1·58; 95% CI, 1·04–2·42) in DMPA users; however, expression levels of two of the main drivers of the inflammatory response were significantly decreased in DMPA users compared to Controls: IL1beta (mean ratio, 0·31; 95% CI, 0·13–0·73) and IL6 (mean ratio, 0·39; 95% CI, 0·21–0·72). Expression levels of other pro-inflammatory (IFNgamma, IL12, tumor necrosis factor (TNF)-alpha) and immune suppressive (IL10) cytokines were not statistically significantly different between groups; however, higher age was significantly associated with lower expression of IL12 and TNFalpha (*P*<0.05).

When examined by duration of DMPA use (none, <30 months [*n*=10], ≥30 months [*n*=4]), the age-adjusted effects of DMPA use on MCP1, IL1beta, and IL6 remained biologically (ratio≥1.5 or <1/1.5) and statistically (*P*<0.05) significant; the effect on IFNa2 became non-significant (*P*=0.15); and the effect on IL10 became significant, reflecting a large decrease among longer-term users (*P*=0.003). When examined by DMPA use since time of last injection (never, ≥30 days ago [*n*=6], <30 days ago [*n*=8]), the age-adjusted effects of DMPA use on MCP1 and IL6 remained significant at *P*<0.05, while IL1b and IFNa2 became less significant (0.05<*P*≤0.11) further from the time of injection, and no other biomarker was statistically significant.

### Effects of DMPA on the phenotypes of endocervical and endometrial T cells

**Figure 2:**
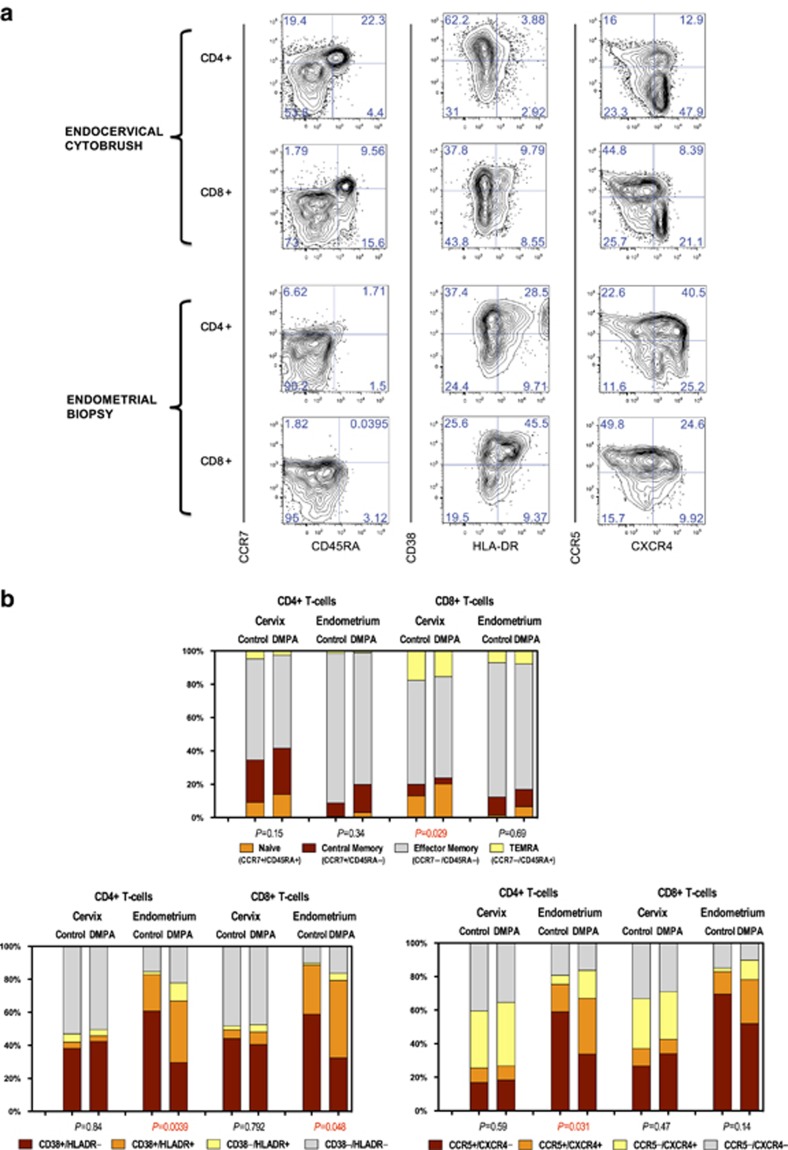
(**a**) Flow cytometry gating used in the analysis of endocervical and endometrial T-cell phenotypes. After designating a lymphocyte gate based on scatter, followed by doublet discrimination, dead cells were removed by dye exclusion; CD3+CD66b− viable cells (not shown) were then subdivided into CD4+ or CD8+ populations. The resulting T-cell populations were then assessed for expression of three pairs of surface markers, as described in the text. Shown from left to right, these were: differentiation markers CCR7 and CD45RA; activation markers CD38 and HLA-DR; chemokine receptors CCR5 and CXCR4. Quadrant gates were drawn based on fluorescence-minus-one (FMO) controls. Numbers in each quadrant indicate percentages of CD4+ or CD8+ T cells expressing various combinations of markers. Data shown are from a representative participant using DMPA. (**b**) Flow cytometric phenotyping data. Bivariate distributions of three pairs of phenotypes: (i) CCR7/CD45RA, (ii) CD38/HLADR, and (iii) CXCR4/CCR5, are summarized for CD4+ and CD8+ T cells from endocervical cytobrush and endometrium, as indicated in the headings. The stacked box plots summarize the relative proportions of each phenotype differentiated by color, analyzed as a function of exposure group (control or DMPA) in models stratified by tissue and cell type. *P*-values are reported for distributional differences between arms as described in Methods section; those values <0.05 are shown in red. Color-coding for each pair of phenotypic markers follows the same pattern in all graphs: +/− red, −/+ yellow, +/+ orange, −/− gray. TEMRA indicates terminally differentiated effector cells expressing CD45RA.

To determine whether DMPA use was associated with changes in T-cell immune characteristics, the surface markers on CD4+ and CD8+ T cells collected via endocervical brushings (14 DMPA users, 25 Controls) and endometrial biopsies (10 DMPA users, 16 Controls) were analyzed by flow cytometry. Representative gating used in the analysis of endocervical and endometrial T-cell phenotypes is shown in Figure 2a.

Effector memory T cells (CCR7-/CD45RA-) were the dominant subset in both endocervix and endometrium. These cells secrete effector cytokines and chemokines and are important for host defense against infectious agents such as HIV. In Controls, a significantly higher percentage of CD4+ and CD8+ T cells had an effector memory phenotype in endometrium compared to endocervix (CD4: mean 89.1 vs. 59.7% CD8: mean 78.9 vs. 61.2%), confirming previous studies (Figure 2b).^22^ In DMPA-treated women, the proportions of effector memory CD4+ and CD8+ T cells in both tissues were reduced compared to control women, but not significantly. Thus, DMPA treatment did not appear to have a major impact on effector memory T-cell subsets in either tissue. Some changes in T-cell maturation phenotype were noted, however: in the endocervix, the proportion of CD8+ naïve cells (CCR7+CD45RA+) was slightly higher in DMPA users than Controls (+7.2%); in the endometrium, the proportion of CD4+ naïve plus central memory cells (CCR7+CD45RA+, CCR7+CD45RA-) was higher in DMPA users (+10.9%).

T-cell activation markers (CD38 and HLA-DR) showed distinct patterns of expression on endometrial compared to endocervical T cells (Figure 2b). In Controls, a significantly higher proportion of endometrial CD4+ and CD8+ T cells expressed CD38 compared with endocervical T cells (CD4: mean 82.8 vs. 43.0% CD8: mean 82.7 vs. 44.3%), confirming previous studies.^22^ In DMPA users, the proportions of endometrial T cells expressing HLADR (CD38+HLADR+, CD38-HLADR+) were higher compared to controls (CD4+, +24.3% CD8+, +20.2%), whereas the proportions expressing CD38 alone (CD38+HLADR-) were lower (CD4+, −31.1% CD8+, −26.4%).

Chemokine receptors CXCR4 and CCR5 also had different patterns of expression on endometrial compared with endocervical T cells (Figure 2b). In control women, a significantly higher proportion of endometrial CD4+ and CD8+ T cells expressed CCR5 compared with endocervical T cells (CD4: mean 69.0 vs. 24.9% CD8: mean 73.5 vs. 36.4%), confirming previous studies.^22^ Finally, the proportions of T cells expressing CXCR4 (CXCR4+CCR5+, CXCR4+CCR5-) were higher among DMPA users (CD4+, +28.2% CD8+, +22.2%), whereas smaller proportions of T cells expressed CCR5 alone (CXCR4-CCR5+) among users than controls (CD4+, −25.2% CD8+, −17.5%).

### Effects of DMPA on densities of immune cells in the endometrium

**Figure 3:**
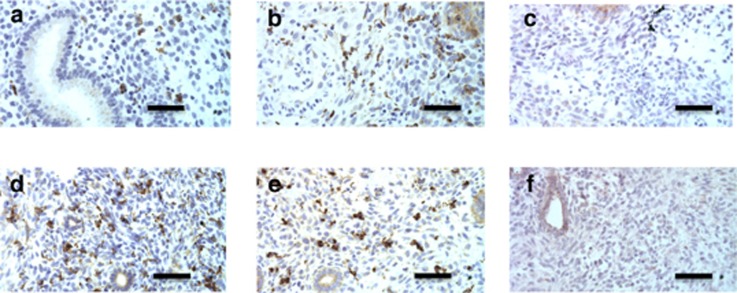
The figure demonstrates differences in macrophage density by immunohistochemical detection in endometrial biopsies from 2 control participants (**a** and **b**) and 2 DMPA users (**d** and **e**). Five-micron paraffin sections of formalin-fixed endometrial biopsies were incubated with mouse monoclonal anti-CD68 antibodies (**a**, **b**, **d** and **e**). Background staining was measured by incubation with an immunoglobulin IgG1 isotype control (1:50) matching the anti-CD68 isotype (**c** and **f**). Detection of antibody binding was measured by peroxidase-conjugated goat anti-mouse secondary antibodies and diaminobenzidine substrate, as described in Methods section. Slides were counterstained with hematoxylin. Dark brown nuclear staining reflects the presence of macrophages. Bars=50 μm.

To determine whether the quantity of key immune cell types in endometrium differed between DMPA users and Controls, we performed IHC on endometrial biopsy sections using markers for natural killer (NK) cells (CD56), macrophages (CD68), CD8+ T cells, and regulatory T cells (FoxP3). Because DMPA use thins the endometrial lining, the amount of tissue obtained for immunohistochemistry was insufficient for CD4 staining. The densities of NK cells and CD8+ T cells among DMPA users (*n*=10) were comparable to Controls (*n*=12; Table 2). However, the density of macrophages was significantly increased (2.05-fold, *P*=0·04), whereas that of regulatory T cells was significantly decreased (0.40-fold, *P*=0·016). Representative images of macrophage immunohistochemistry are presented in Figure 3.

## Discussion

CD4+ CCR5+ T cells are major targets for HIV infection; hence their presence in the reproductive tract is an important surrogate for HIV susceptibility. In control women, the proportion of CD4+ T cells expressing HIV co-receptors CXCR4 and CCR5 was significantly higher in the endometrium compared to the endocervix, as was the proportion of activated T cells. These findings suggest a potential increased susceptibility to HIV infection in the endometrial compared with the endocervical compartment, confirming previous findings^22^ and reinforcing recent attention on the upper FRT as a possible portal for HIV acquisition and/or reservoir for viral replication and dissemination.^20^

CCR5 expression was lower on endometrial T cells in DMPA users compared to control women, suggesting that observed effect of DMPA in enhancing HIV acquisition risk is not mediated by increased CCR5 expression in the endometrium. Conversely, the proportions of endometrial CD4+ and CD8+ T cells expressing markers of T-cell activation were higher in DMPA users than in control women, providing a potential mechanism for enhanced risk.

In the endocervix, others have shown that the frequency of activated CD4+ CCR5+ T cells was increased in DMPA users and in the luteal phase (time of peak progesterone level) among women not using contraceptives compared to the follicular phase, implicating high progestin states (endogenous or exogenous) as biological modifiers of HIV target cell density at the cervix.^16^ In our study, samples from controls were collected at peak progesterone levels (the luteal phase), and showed no difference in CD4+ CCR5+ T-cell density compared with DMPA users.

Our results also highlight important differences between the lower and upper reproductive tract. Although we found no significant difference between samples from DMPA users and control women in the frequency of cervical CD4+ CCR5+ T cells and T-cell activation status, in a study by Chandra *et al.*^6^ of women 12 weeks after a single DMPA injection, the density of vaginal CCR5+ cells was significantly increased compared with samples collected from the follicular or luteal phases of those women before they received the injection. The differences between the vaginal and endocervical findings support *in vitro* findings^7^ that progestins have differential effects on cellular compartments in the reproductive tract, highlighting the importance of studying the cervix and upper female reproductive tract to characterize fully the effects of contraceptives on HIV risk.

Another effect of DMPA on immune cells in the upper FRT that we observed was an increased density of macrophages in the endometrium. MCP1, a chemokine expressed by epithelial and inflammatory cells that recruits macrophages to sites of inflammation was significantly increased in endocervical fluid from DMPA users. Macrophages with an M2-like phenotype (CD68+/CD163+/CD206+/IL-10^high^) have recently been shown to be the primary target of HIV infection in the endometrium.^23^ We were not able to characterize the phenotype of the macrophages due to the scant volume of endometrial tissue available for analysis from DMPA users, and hence we do not know if the observed increase in macrophage density included the susceptible phenotype. However, of note, our findings are consistent with transcriptional profiling of endometrial samples from participants of this study, in which the endometrial transcriptome in DMPA users was characterized by marked upregulation of pathways regulating cell movement of myeloid cells, cell movement of phagocytes, and adhesion of immune cells.^24^

Prolonged exposure to DMPA results in quiescence or atrophy of the endometrium, characterized by thinning of the epithelium, inactive widely spaced glands, stromal edema, and pseudodecidualization.^25, 26, 27^ These morphological changes may account for our observed alterations in cellular composition in DMPA users, rather than the hormonal effects of DMPA itself. Comparison with samples from postmenopausal women, who are also atrophic but not exposed to DMPA, would address this issue, but was not a goal of this study. The median length of DMPA exposure was 13 months in our study, a time at which all our endometrial specimens would be predicted to be at least quiescent if not frankly atrophic.^25, 26, 27^

DMPA was associated with changes in the immune microenvironment of the endocervical canal, specifically with decreased levels of IL6 and IL1beta. In cultured endocervical cells, MPA has immunosuppressive effects, signaling predominantly through the glucocorticoid receptor and resulting in decreased production of IL6 and other inflammatory mediators.^28^ MPA added to peripheral blood mononuclear cells also suppressed expression of IL6, IL1beta and other factors.^9^ Our findings in endocervical fluid are therefore consistent with observed *in vitro* immunosuppressive effects of MPA on both endocervical cells and infiltrating immune cells, and could result in impaired protection against HIV infection in the endocervix.

Our results differ from two studies in Africa that showed an increase in RANTES in endocervical samples from DMPA users, which we did not observe.^29, 30^ One of those studies found increased levels of IL6, whereas we observed decreased levels.^30^ These differences may be due to the populations studied, data analysis methods, and/or to methods used for specimen collection: we used a sponge wick inserted into the endocervical canal for 90 s, whereas the other studies used a swab inserted into canal and gently rotated for 3–5 s. Thus it is possible that these different collection techniques sampled different compartments of the endocervix. Our data analysis methods may have increased our discriminatory power.

Strengths of our study include measurement of a wide range of immune parameters and the use of samples from Controls timed to a narrow window of peak progesterone levels, thereby minimizing the effects of endogenous hormone fluctuations on the measured parameters. In addition, the samples from DMPA users were collected in women who had used the method for >6 months, thereby reflecting effects of prolonged exposure. Finally, analyses of endocervical cytokines and chemokines that accounted for recency of DMPA injection and for duration of DMPA use generally supported our results.

Limitations of our study include the relatively small numbers of samples analyzed and statistically but not biologically significant differences in age between the Control and DMPA groups, We selected the age range of participants to reflect a hormonally stable time frame, avoiding fluctuations associated with menarche and perimenopause. Further, age-adjusted analyses found that increased age is associated with lower levels of only three endocervical biomarkers, none of which differed significantly by study group. Another limitation is that prolonged exposure to DMPA results in endometrial epithelial thinning, resulting in small amounts or no tissue available for analyses.^25, 26, 27^

Our study documents the effects of DMPA on potential sites of vulnerability to HIV infection in 2 upper FRT sites (endocervix and endometrium), and documents important differences in DMPA effects in the upper tract with published results from the lower tract. Suppression of the local immune response in the endocervix may enhance risk of HIV acquisition by reducing the local defenses against viral infection. Our observation of increased macrophage density in the endometrium of DMPA users might offer a mechanism to explain the observed association with HIV acquisition. Further research is needed to determine effects of DMPA use on HIV infection of target cells in both the upper and lower reproductive tracts, and whether these effects explain the observed association with increased risk of HIV infection.

## Methods

### Study design.

This was a cross-sectional study comparing women using DMPA with women using no hormonal or intrauterine contraception (Controls). We have previously reported on the effects of 2 other contraceptives on the upper FRT: nonoxynol-9 (see ref. 31) and the levonorgestrel-releasing intrauterine device.^24, 32^ The Control group of women not on hormonal contraceptives used for the current study of DMPA effects is the same as the Control group that was used for the LNG-IUD study.^24, 32^ The UCSF Human Research Protection Program & IRB approved the study protocol, recruiting and consent materials (approval #10-01003).

### Recruitment of human volunteers and clinical study procedures.

 Procedures for participant recruitment, screening and enrollment were performed as previously described.^31, 32^ Briefly, exclusion criteria included: age <18 or ≥45 years, positive HIV serology, positive urine nucleic acid amplification test for *Neisseria gonorrhoeae* or *Chlamydia trachomatis*, or current or recent pregnancy or breastfeeding. Participants agreed to refrain from using vaginal products (creams, douches) for at least ten days prior to study biopsies, to use non-lubricated condoms throughout the study period, and to abstain from any vaginal intercourse for 72 h prior to biopsy procedures. Controls reported no use of exogenous hormones for at least three months and a history of at least three normal and consecutive menstrual periods since discontinuation. DMPA users were required to have used that method for at least six months. Controls were instructed to measure their urine for luteinizing hormone (LH) detection as previously described,^31, 32^ and to come in for collection of study specimens 7–11 days after the LH surge so that their study visit occurred in the luteal phase when endogenous progesterone levels were high.

For specimen collection, a speculum was inserted into the vagina, the cervix was visualized, and the following specimens were collected: endocervical fluid via wick (an ophthalmic sponge Merocel eye spears, Beaver Visitec International, Waltham, MA, USA); endocervical sample using an endocervical cytobrush (Cytobrush Plus Cell collector, CooperSurgical, Trumbull, CT 06611) turned three times; a cervical biopsy (performed at the transformation zone if visible or at the os using a Mini-Tischler punch biopsy forceps); and an endometrial biopsy using a three mm cannula (Miltex brand Softflex) inserted through the internal os into the endometrial cavity. Expression profiling of RNA isolated from the cervical biopsy and a portion of the endometrial biopsy for these samples and those from women using a levonorgestrel intrauterine device have been published previously.^24^

### Endocervical wick cytokine/chemokine measurements.

 Endocervical wick samples were snap frozen at the time of collection and stored at −80 °C until analysis in bulk. Wick samples were processed as previously described.^31, 32^ Samples were assayed on the Milliplex panel (Millipore, Billerica, MA, USA) for the following cytokines (with limits of detection in pg ml^−1^): IFNalpha2 (2.9), IFNgamma (0.8), IL10 (1.1), IL12p70 (0.6), IL1alpha (9.4), IL1beta (0.8), IL6 (0.9), IL8 (0.4), MCP1 (1.9), MIP1alpha (2.9), MIP1beta (3.0), RANTES (1.2), and TNFalpha (0.7). All samples were run in duplicate and the overall intra-assay coefficient of variability was 10.0%. A set of 8 samples was run on duplicate plates and the inter-assay variability was 11.5%. The plates were read on a Bio-Plex Suspension Array Reader (Bio-Rad Laboratories, CA).

### Phenotypic analysis of cervical and endometrial T cells.

Blood, endocervical brushings and endometrial biopsies were collected and processed within 4 h as previously described.^22, 31, 32^ Fluorochrome-labeled monoclonal antibodies included CD3 (UCHT1), CD4 (RPA-T4), CD8 (SK1), CCR7 (3D12), CXCR4 (12G5), CCR5 (2D7), CD8 (RPA-T8), from Becton-Dickinson Pharmingen (San Diego, CA); CD45RA (2H4), CD4 (T4D11) from Beckman Coulter (Fullerton, CA); CD38 (HB7) from BD Biosciences (San Jose, CA); HLADR (TU36) and aqua amine dye from Invitrogen (Carlsbad, CA). Optimum titers were determined based on preliminary titration experiments. Fluorescence minus one (FMO) controls were utilized to establish positive cutoffs.^33^ Flow cytometry data were acquired on an LSRII (BD Immunocytometry Systems, San Jose, CA, USA) equipped with 405, 488 and 643 nm lasers and utilizing FACSDIVA software (BDIS). Analysis was done with FlowJo software (TreeStar, Ashland, OR, USA). Results were recorded as the percentage of CD4+ or CD8+ T cells expressing a given marker or combination of markers.

### Immunohistochemistry of endometrium.

At the time of collection, a portion of each endometrial biopsy was fixed in formalin and paraffin embedded. Five-micron sections were stained with mouse monoclonal antibodies against human CD8 (c8/144B, ThermoFisher); CD56, a marker of NK cells (123c3.d5, ThermoFisher); CD68, a marker of macrophages (KP1, Dako, Agilent Technologies Inc, Santa Clara, CA); and FoxP3, a marker of regulatory T cells (123c3.d5, ThermoFisher) using previously published techniques.^32, 34, 35, 36^ Peroxidase-conjugated goat anti-mouse secondary antibodies (Vector Laboratories Inc, Burlingame, CA) and diaminobenzidine substrate (Dako) were used for detection. Positively stained cells were counted on photomicrographs of 5–10 fields from each slide. As the tissue in the photomicrograph did not always fill the field, the area that contained tissue was outlined and measured on a grid; counts represent the number of stained cells divided by the number of grids included in the image. Tissue areas covering more than 96 grids (out of a total possible 382.5 grids) were included in the analysis. To avoid counting projections from a single CD68+ cell as multiple cells, CD68 staining had to be associated with a nucleus to be counted. To be counted as a FoxP3+ cell, the staining had to be nuclear.

### Statistical methods.

#### Participant characteristics:

we present demographic and eligibility characteristics of study participants by exposure group, comparing mean ages via *t*-test and race distributions via Fisher’s exact test. We summarize cycle day among Controls, and days since last DMPA injection and duration of DMPA exposure among DMPA users via medians and interquartile ranges (IQR).

#### Endocervical protein measurements:

Wick sampling yielded one observation per biomarker per participant. Protein concentrations were analyzed as linear functions of exposure group and age (centered at 30 years old) using generalized estimating equation (GEE) models, assuming negative binomial distributions and using a log link and robust standard errors. We report age-adjusted group-specific biomarker concentrations in a forest plot of the corresponding exposure effects, expressed as a mean (95% CI) DMPA:Control relative concentration (ratio), and Wald *P*-values. We also created two 3-level ordinal measures of increasing DMPA exposure by classifying Controls as “not exposed” and classifying DMPA users by either: (i) duration of use (less vs. more than 30 months of use), or (ii) most recent DMPA injection (more vs. less than 30 days before enrollment). We modeled biomarker concentrations as a linear function of each ordinal measure, adjusted for age. We chose to look at time since DMPA injection because pharmacokinetic data suggesting serum MPA levels peak shortly after administration and then rapidly decline.^37^

#### Flow cytometry:

flow cytometry assays, based one endocervical and one endometrial sample per participant, generated person-level joint distributions of three pairs of phenotypes separately for CD4+ and CD8+ cell types: CCR7/CD45RA; CD38/HLADR; and CXCR4/CCR5. To account for repeated measures per participant, component percentages were estimated as a function of age (centered), exposure group, component level, and the group-by-level (SAS genmod, assuming normal distributions) in models stratified by tissue and cell type. We present age-adjusted mean distributions via plots by arm, tissue, and cell type, and report *P*-values that test for distributional differences between arms (3 degree-of-freedom interaction-effect based on empirical standard errors). (Note: identical estimates were obtained by analyzing person-specific distributions as multinomial outcomes, which constrains the component percentages (e.g., of CCR7+/CD45RA+, CCR7+/CD45RA−, CCR7−/CD45RA+, CCR7−/CD45RA-) to sum to 100% (SAS logistic with glogit link)).

#### Immunohistochemical analysis of immune cell densities:

for each specimen per participant, biomarker cell counts were determined in multiple standardized areas (see Methods section for Immunohistochemistry above). Only participants with five to ten qualifying areas were included in the analysis, generating different sample sizes per marker. Counts of each biomarker were analyzed as above (negative binomial distribution, logarithm link, and robust variance estimator) by study group, with an offset used to adjust for the sizes of the areas counted. Mean (95% CI) DMPA:Control differences on the log scale, estimated by the model, were transformed to the natural scale for reporting as ratios (relative concentrations).

Statistical analyses were conducted using SAS version 9.4 (SAS Institute Inc, Cary, NC). All *P*-values cited are two-sided and values less than *α*=0.05 were considered statistically significant.

## Figures and Tables

**Table 1:** Demographic and Eligibility Characteristics by Study Group

**Characteristics**	**Control** ***n*****=27**	**DMPA** ***n*****=15**	***P*****-value**
Age, mean (min, max)	34.6 (23–44)	27.8 (21–38)	0.002^a^
			
Race, *n* (%)			0.13^b^
White	15 (55.6)	6 (40.0)	
Black	9 (33.3)	9 (60.0)	
Asian	3 (12.5)	0 (0.0)	
			
Cycle day, median (IQR)	23 (20, 26)	—	
Days since last DMPA injection, median (IQR)	—	28.5 (13, 77)	
Months of DMPA exposure, median (IQR)	—	15 (13, 33)	

Abbreviations: IQR, interquartile range; DMPA, depot-medroxyprogesterone acetate.

a Student’s *t*-test.

b Fisher’s exact test.

**Table 2:** Quantification of cell types by immunohistochemistry in endometrial biopsy specimens ordered by decreasing density of cell types among Controls, based on samples from 12 Controls and 10 DMPA users

**Antibody (cell type)**	**Control** **Mean cell density**^a^ **(95% CI)**	**DMPA:Control** **Fold change (95% CI)**	***P*****-value**^b^
CD56 (NK cell)	71.2 (30.0,168.7)	2.08 (0.70,6.20)	0.19
CD68 (macrophage)	55.4 (38.0,80.6)	**2.05 (1.03,4.07)**	**0.04**
CD8 (T cell)	18.8 (11.5,30.8)	1.66 (0.78,3.53)	0.19
FoxP3 (regulatory T-cell)	5.54 (3.54,8.68)	**0.40 (0.19,0.40)**	**0.016**

Abbreviations: CI, confidence interval; DMPA, depot-medroxyprogesterone acetate. NK cells, natural killer cells.

a Density is defined as the number of positively stained cells in a standardized unit of area from photographic images of × 40 microscopic fields.

b Results that are statistically significant with *P* value <0.05 are shown in boldface.
